# Irisin as an Associative Marker of Metabolic Dysregulation in Obesity: Comparative Profiling of IL-6, IL-15, IL-1β, and CCL2

**DOI:** 10.3390/diagnostics16101459

**Published:** 2026-05-11

**Authors:** Wiktoria Narloch, Marta Jaskulak, Klaudia Antoniak-Pietrynczak, Katarzyna Zorena

**Affiliations:** 1 Environment and Health Scientific Circle, Department of Immunobiology and Environment Microbiology, Medical University of Gdańsk, 80-210 Gdańsk, Poland; 2 Department of Immunobiology and Environment Microbiology, Faculty of Health Sciences, Medical University of Gdansk, 80-211 Gdańsk, Poland; klaudia.antoniak@gumed.edu.pl (K.A.-P.); kzorena@gumed.edu.pl (K.Z.)

**Keywords:** overweight, obesity, adipokines, cytokines, PCA, CCL2, irisin

## Abstract

**Background/Objectives:** Obesity is a complex metabolic disorder associated with chronic low-grade inflammation, insulin resistance, and increased risk of metabolic complications. Traditional measures, such as body mass index (BMI), may not detect early metabolic disturbances. Myokines and cytokines, including irisin, C-C Motif Chemokine (CCL2), interleukin-1β (IL-1β), interleukin-6 (IL-6), and interleukin-15 (IL-15), have been proposed as potential biomarkers. This study aimed to investigate the relationships between these biomarkers and metabolic parameters in adults with varying BMI. **Methods:** Fifty-one adults (mean age 38 ± 11 years) were stratified by BMI into normal weight, overweight, and obese groups. Serum irisin, CCL2, IL-1β, IL-6, and IL-15 concentrations were measured along with metabolic parameters, including insulin, HOMA-IR, C-peptide, and visceral fat. Statistical analyses included Pearson’s correlation, Kruskal–Wallis ANOVA with Bonferroni correction, and subgroup analyses for insulin resistance (HOMA-IR ≥ 2.5). **Results:** Irisin concentrations were significantly higher in overweight and obese participants compared with normal-weight individuals (*p* < 0.001) and positively correlated with insulin (r = 0.77), HOMA-IR (r = 0.63), C-peptide (r = 0.71), and BMI (r = 0.54). In contrast, IL-6 and IL-15 levels did not differ significantly across BMI groups, although IL-15 showed a borderline increase in insulin-resistant individuals (*p* = 0.048). Both IL-1β and CCL2 were significantly elevated across increasing body-weight categories and showed strong positive correlations with measures of adiposity, visceral fat, and insulin resistance; however, neither marker differed significantly when participants were stratified by insulin-resistance status. Additionally, multivariable linear regression identified irisin as the only independent predictor of insulin resistance, while CCL2 was the strongest predictor of BMI. Principal component analysis (PCA) revealed two dominant components separating metabolic (irisin, HOMA-IR, insulin, BMI) and inflammatory (IL-1β, CCL2) profiles, supporting the distinction between metabolic and inflammatory pathways in obesity. **Conclusions:** Irisin appears to be a sensitive associative marker of metabolic dysregulation associated with increased body mass and insulin resistance. In contrast, IL-1β and CCL2 reflect obesity-related inflammatory burden rather than early metabolic changes, while IL-6 and IL-15 did not reflect early metabolic alterations in this study. Together, these findings suggest that irisin may serve as an associative biomarker for identifying individuals at risk of obesity-related metabolic disturbances, whereas CCL2 and IL-1β may be more indicative of chronic adipose tissue inflammation.

## 1. Introduction

Obesity is a global health issue that has reached epidemic proportions due to its widespread prevalence and the number of individuals affected by the condition. According to the World Health Organization, in 2022, 2.5 billion adults were overweight, of which 890 million were obese. At the same time, over 390 million children and adolescents aged 5 to 19 were overweight, and 160 million of them were living with obesity [1]. Traditional methods for assessing obesity, such as the Body Mass Index (BMI), do not always accurately reflect the complexity of this condition, as it lacks precision and does not account for individual differences; thus, it is mainly used to assess the health status of entire populations [2,3]. This, along with the growing number of individuals affected by obesity, drives researchers to continuously seek more accurate diagnostic tools. As a result, interest in biomarkers of worsening metabolic status including associative biomarkers such as interleukin-6 (IL-6), interleukin 15 (IL-15), interleukin 1-β (IL-1β), monocyte chemoattractant protein-1 (CCL2), and irisin which demonstrate statistically significant associations with the presence of specific diseases, health conditions, or environmental exposures, and may therefore be applied in diagnosis and in monitoring treatment outcomes is growing [3,4]. New associative biomarkers could provide more detailed insights into the pathophysiology of obesity and its associated health risks. Additionally, the presence of specific biomarkers may help characterize the type of obesity and its related health consequences [4]. Given that obesity is associated with chronic inflammation closely linked to metabolic disturbances such as insulin resistance and type 2 diabetes, there is growing interest in the role of cytokines and myokines as key biomarkers of communication between the immune system, skeletal muscles, and energy regulation in the body. Among them, particular attention has been paid to interleukin-6 (IL-6), interleukin-15 (IL-15), and irisin—a myokine released from muscles in response to physical activity, derived through proteolytic cleavage of the FNDC5 protein [5]. Growing interest has also been focused on interleukin-1β (IL-1β), which is one of the key pro-inflammatory cytokines and has been associated with the induction of insulin resistance, pancreatic β-cell dysfunction, and the amplification of inflammation within adipose tissue, a hallmark of obesity [6]. IL-1β is also one of the major pro-inflammatory cytokines secreted by adipose tissue macrophages during the development of obesity. With the progression of obesity, macrophages undergo a shift toward a pro-inflammatory phenotype, accompanied by increased expression of IL-1β, along with TNF-α and IL-6, a phenomenon observed mainly in murine models. This cytokine is particularly highly produced by the population of CD11c^+^ macrophages present in the adipose tissue of obese individuals. Importantly, IL-1β is also secreted by macrophages exhibiting a mixed, metabolically activated phenotype, which combines features of both M1 and M2 cells, highlighting its central role in sustaining chronic inflammation in obesity [7]. Another important marker of metabolic disturbances in obesity is the chemokine CCL2 (monocyte chemoattractant protein-1, MCP-1), which is responsible for the recruitment of monocytes and macrophages into adipose tissue, thereby promoting the maintenance of chronic inflammation [8]. Dommel and Blüher (2021) reported murine studies demonstrating that increased CCL2 expression in adipocytes, associated with insulin resistance, leads to adipose tissue inflammation, whereas deficiency of the CCR2 receptor is linked to reduced macrophage infiltration and improved insulin sensitivity under a high-fat diet [8].

Each of these associative biomarkers plays a significant yet complex role in the regulation of inflammatory processes, energy homeostasis, and glucose and lipid metabolism. Irisin exhibits anti-inflammatory properties, and studies on mice suggest that it may reduce the production of pro-inflammatory cytokines such as IL-6 and TNF-α by inhibiting signaling pathways including NF-κB and supporting the polarization of macrophages toward the anti-inflammatory M2 phenotype [9,10]. Considering irisin’s role in modulating inflammatory pathways and its association with improved metabolic parameters, exploring its therapeutic potential may prove highly valuable in the context of the growing obesity problem worldwide. IL-15, on the other hand, is a cytokine that plays a key role in muscle function and metabolic regulation. Research indicates that it may influence insulin sensitivity and body fat composition. Although a direct relationship between IL-15 and insulin resistance has not yet been clearly established, its involvement in signaling between muscles and adipose tissue suggests its potential significance in maintaining metabolic health [11]. Moreover, there is evidence suggesting that interactions between IL-15, IL-6, and irisin play a crucial role in the regulation of metabolic processes that are fundamental to the development and progression of obesity [12]. Each of these myokines affects the metabolism of both adipose and muscle tissue individually, but their synergistic action and communication between these two tissue types may play a key role in maintaining the body’s energy balance [13]. IL-6 and irisin are known to promote the browning of white adipose tissue and increase thermogenesis [14], whereas IL-15 supports fat mass reduction and enhances mitochondrial function by reducing oxidative stress and lipid accumulation, as demonstrated in mouse models [15]. Taken together, the aim of this study is to evaluate the combined effects and interactions of these molecules in the context of metabolic health in individuals with obesity, as understanding their role may lead to a more accurate grasp of the underlying mechanisms of this condition and contribute to the development of more effective diagnostic and therapeutic strategies.

## 2. Materials and Methods

### 2.1. Study Population

The inclusion criteria were (1) only adults (age > 18 years); (2) impaired glucose tolerance; (3) insulin resistance and/or hyperinsulinemia; (4) not taking medications able to modify glucose metabolism (e.g., including antipsychotics, cortisone, diuretics, transcriptase or protease inhibitors) in the previous 3 months; (5) no changes in dietary regimen within the previous 3 months; (6) no changes in physical activity within the previous 3 months; (7) BMI from 20 to 35 kg/m^2^. The exclusion criteria included (1) diagnosis of T1DM/T2DM; (2) uncontrolled arterial hypertension; (3) clinically relevant cardiac arrhythmia; (4) suffering from deep and superficial vein thrombosis; (5) acute kidney injury and liver failure; (6) presence of neoplastic diseases; (7) presence of autoimmune diseases; (8) very physically active life-style, resistance training, (9) infectious diseases. All of the patients received oral and written information about the study, and written informed consent forms were obtained prior to enrolment in the study. Biochemical parameters, including fasting glucose, lipid profile (total cholesterol, LDL, HDL, triglycerides), insulin, C-peptide, and C-reactive protein (CRP), were measured from venous blood samples collected from all participants under standardized conditions (morning collection after an overnight fast). Blood samples were processed according to routine clinical laboratory procedures in a certified diagnostic laboratory. For the analysis of cytokines, the biological material analyzed consisted of peripheral blood serum, collected under standard conditions (fasting, in the morning hours). Study group consisted of participants with a mean age: 38 ± 11 totaling 51 individuals (female). Samples were stored at −80 °C until analysis to prevent degradation of proteins and bioactive compounds. The study group was divided into three groups: individuals with normal body weight (BMI: 18.5–24.9 kg/m^2^), overweight individuals (BMI: 25.0–29.9 kg/m^2^), and obese individuals (BMI: ≥30.0 kg/m^2^). Table 1 presents the mean values of the measured parameters for each group. The Tanita SC-240 foot-to-foot body composition analyzer (Tanita Corporation, Tokyo, Japan) was used to assess bioelectrical impedance. All measurements were collected at 50 Hz using the standard setting after manually inputting the measured gender, age, and height of the patient. The patients wore minimal clothing, no jewelry, were barefoot, and were commanded to stand still with their feet in direct contact with all four metal plates. The level of the visceral adipose tissue was calculated using the internal equations of the Tanita analyzer.

The study was approved by the Bioethics Committee of the Medical University of Gdańsk (approval no. NKBBN/692/2019-2020; approval date: 30 January 2020). All participants provided written informed consent prior to participation.

### 2.2. Quantitative Measurement of IL-6, IL-15, IL-1β, CCL2 and Irisin Concentrations

Five sandwich ELISA tests were performed to quantitatively determine the concentrations of IL-6, IL-15, IL-1β, CCL2, and irisin in serum samples from all study participants. The analysis was conducted using kits from R&D Systems (Minneapolis, MN, USA) and the intra- and inter-assay coefficients of variation (CV) were provided by the manufacturer (R&D Systems) and were determined according to the manufacturer’s protocol. Intra-assay precision was assessed by testing three samples of known concentration on a single plate, with each sample measured twenty times. Inter-assay precision was determined by testing the same three samples in twenty separate assays: a Human IL-15 Quantikine ELISA Kit, catalog no. D1500, where intra-assay precision yielded CV values 3.9%, 5.3% and 3.4% and inter-assay precision yielded CV values 9.1%, 8.2% and 6.6%; a Human IL-6 Quantikine ELISA Kit, catalog no. D6050B, where intra-assay precision yielded CV values of 1.8%, 2.1%, and 2.3% and an inter-assay precision yielded CV values of 4.2%, 3.8%, and 4.3%; a Human IL-1 beta/IL-1F2 Quantikine ELISA Kit catalog number DLB50, where intra-assay precision yielded CV values of 8.5%, 3.3% and 4.4% and inter-assay precision yielded CV values of 8.4%, 4.2% and 4.1%; a Human CCL2/MCP-1 ELISA Kit catalog no DCP00, where intra-assay precision yielded CV values of 7.8%, 4.7% and 4.9% and inter-assay precision yielded CV values of 6.7%, 5.8% and 4.6%; and a Human Irisin/FNDC5 DuoSet ELISA catalog no DY9420-05, where intra-assay precision yielded CV values of 3.5%, 3.3% and 5.4% and inter-assay precision yielded CV values of 6.2%, 4.2% and 6.1%. These tests were selected due to their high sensitivity, precision, and broad measurement range. The entire procedure was carried out according to the manufacturer’s instructions. The results were read using a ChroMate 4300 ELISA microplate reader (Awareness Technology, Inc., Palm City, FL, USA) at a wavelength of 450 nm for each parameter analyzed. All measured concentrations fell within the analytical measurement ranges specified by the manufacturer for each assay. No samples exceeded the upper limit of quantification; therefore, no additional dilutions were required. All analyses were performed using the assay diluents provided by the manufacturer and in accordance with the recommended protocols.

### 2.3. Statistical Analysis

**Figure 1:**
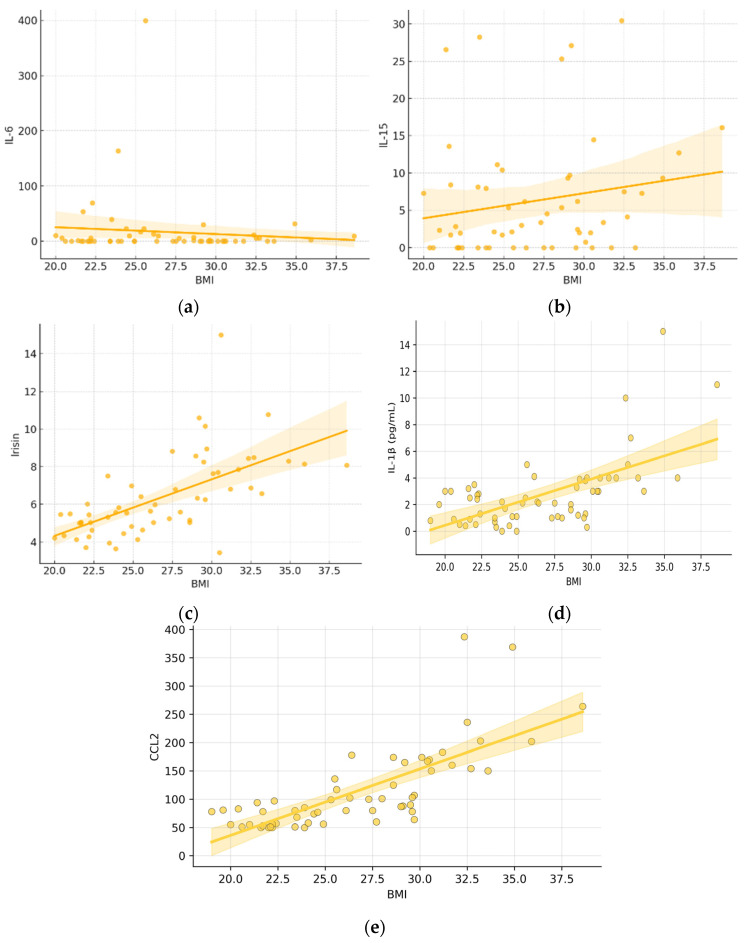
Correlation Analysis. Scatter plots with fitted regression lines showing the correlations between BMI and circulating levels of IL-6 (**a**), IL-15 (**b**), irisin (**c**), IL-1 β (**d**) and CCL2 (**e**). Each dot represents an individual participant. Shaded areas indicate the 95% confidence intervals of the regression lines. BMI—Body Mass Index. The concentrations of IL-6 (**a**), IL-15 (**b**), irisin (**c**), IL-1 β (**d**) and CCL2 (**e**) are expressed in picograms per milliliter (pg/mL).

**Figure 2:**
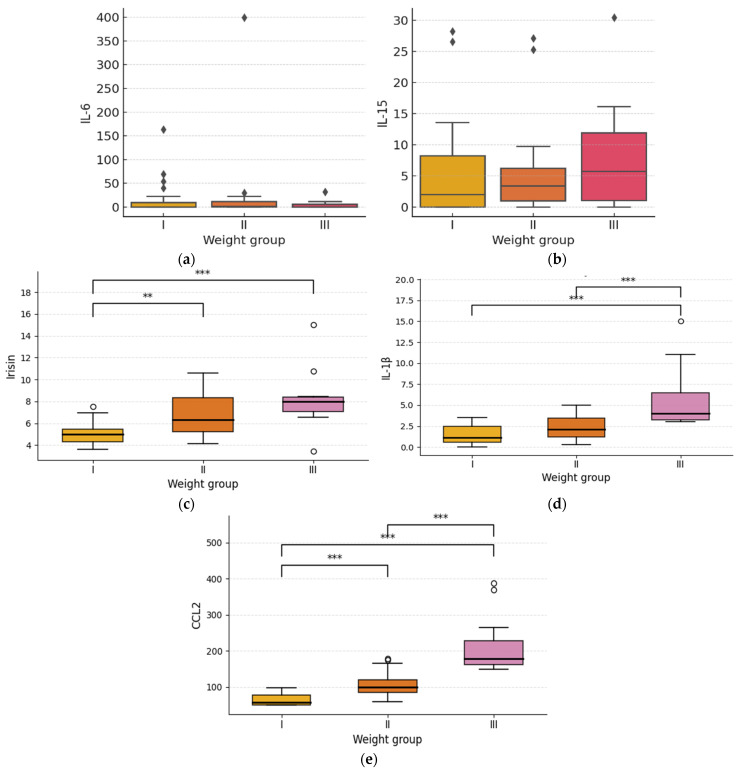
Distribution of IL-6 (**a**), IL-15 (**b**), irisin (**c**), IL-1 β (**d**) and CCL2 (**e**) concentrations among participants categorized into three weight groups: normal weight (I), overweight (II), and obese (III). Each boxplot represents the median, interquartile range (IQR), and outliers (values beyond 1.5 × IQR). I—individuals with normal body weight (BMI: 18.5–24.9 kg/m^2^); II—overweight individuals (BMI: 25.0–29.9 kg/m^2^); III—obese individuals (BMI: ≥30.0 kg/m^2^). Boxplots showing circulating concentrations of IL-6, IL-15, irisin, IL-1β, and CCL2 across weight groups (I–III) in three independent groups. Boxes represent the interquartile range with median values indicated by bold horizontal lines; whiskers denote the minimum and maximum values excluding outliers. Group comparisons were performed using the Kruskal–Wallis test followed by pairwise Mann–Whitney U tests with Bonferroni correction. Significant pairwise differences are indicated by asterisks (* *p* < 0.05, ** *p* < 0.01, *** *p* < 0.001). Across all groups, irisin, IL-1β, and CCL2 showed consistent and significant group-dependent differences, whereas IL-6 and IL-15 did not differ significantly between groups. All values are presented as mean differences expressed in picograms per milliliter (pg/mL).

**Figure 3:**
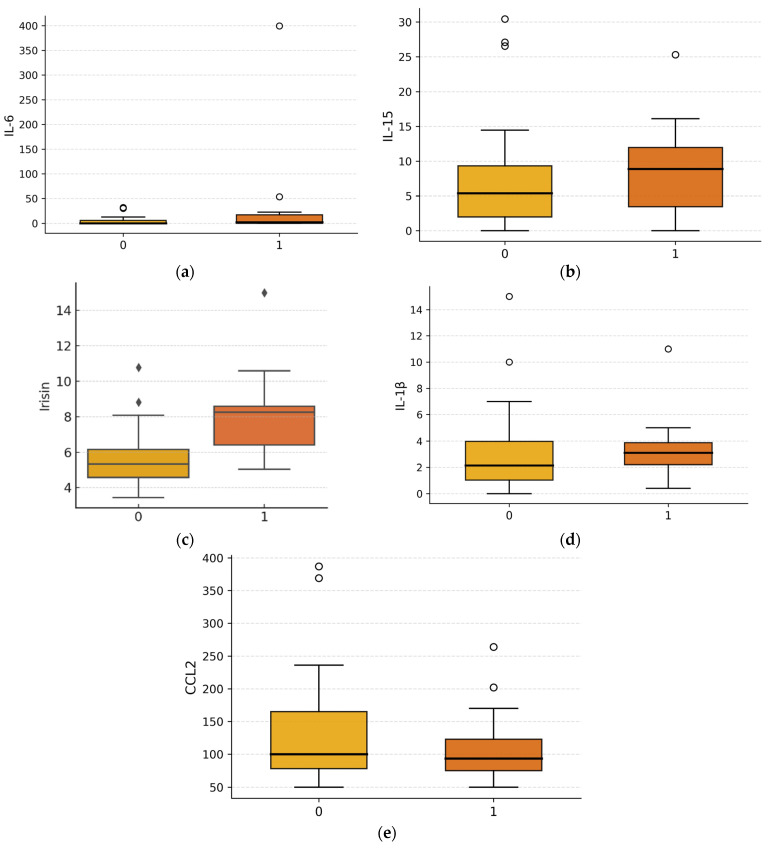
ANOVA analysis in groups stratified by insulin resistance. Boxplots illustrate the distribution of IL-6 (**a**), IL-15 (**b**), irisin (**c**), IL-1β (**d**), and CCL2 (**e**), concentrations in patients without insulin resistance (Group 0) and those with insulin resistance (Group 1). Each box represents the interquartile range (IQR), with the horizontal line inside the box indicating the median value. Whiskers denote data within 1.5 × IQR, and points beyond this range are plotted as outliers. All values are presented as mean differences expressed in picograms per milliliter (pg/mL).

Statistical analysis was performed using OriginPro 2021 software (OriginLab Corporation, Northampton, MA, USA). In the preliminary stage, both Pearson’s and Spearman’s correlation coefficients were calculated to determine the strength and direction of relationships between the investigated metabolic and biochemical parameters (including IL-1β, CCL2, IL-6, IL-15, irisin, insulin, HOMA-IR, C-peptide, BMI, WHR, visceral fat, blood pressure, lipid profile, and CRP) (Figure 1). The obtained correlation coefficients are presented in tabular form along with an assessment of their statistical significance. The corresponding results are summarized in Table 2. Subsequently, differences between groups defined according to BMI values for each participant (normal weight, overweight, obesity) were analyzed. Group comparisons were conducted using the Kruskal–Wallis ANOVA test (Figure 2). Additionally, subgroup analyses were performed taking insulin resistance into account (HOMA-IR < 2.5—absence of insulin resistance; HOMA-IR ≥ 2.5—presence of insulin resistance) (Figure 3). A HOMA-IR cut-off value of 2.5 was applied, as this threshold is commonly used to define insulin resistance in European populations. However, it should be noted that HOMA-IR cut-offs may vary depending on population characteristics, including ethnicity, age, and metabolic status. Comparisons were conducted using the Mann–Whitney U test. For all analyses, the significance level was set at α = 0.05. Values of *p* < 0.05 were considered statistically significant, whereas *p* < 0.001 were considered highly significant. To evaluate the independent associations between circulating biomarkers and metabolic parameters, multivariable (multiple) linear regression analyses were performed. Two separate models were constructed using HOMA-IR and BMI as dependent variables. Independent variables included irisin, IL-6, IL-15, IL-1β, and CCL2. In the HOMA-IR model, BMI was additionally included as a covariate to account for adiposity as a potential confounding factor. Predictor selection was based on biological relevance and prior univariate analyses. Given the sample size, all variables were included simultaneously in a single model per outcome to evaluate their relative contributions. Standardized regression coefficients (β), 95% confidence intervals (CI), and *p*-values were reported. Model fit was assessed using the coefficient of determination (R^2^) and adjusted R^2^.

Assumptions of linear regression, including linearity, homoscedasticity, and normality of residuals, were evaluated using diagnostic plots. Multicollinearity was assessed using variance inflation factors (VIFs), and no significant collinearity was observed. Principal component analysis (PCA) was performed as an exploratory multivariate technique to assess the underlying structure of relationships among circulating biomarkers and metabolic parameters. The analysis included irisin, IL-6, IL-15, IL-1β, CCL2, BMI, HOMA-IR, insulin, and C-peptide. All variables were standardized (z-scores) prior to analysis to ensure comparability. Components were extracted based on eigenvalues >1 (Kaiser criterion), and the proportion of explained variance was calculated. Factor loadings were examined to determine the contribution of each variable to the principal components. PCA results were visualized using a biplot representing both variable loadings and the distribution of observations. Statistical analyses were performed using R version 4.6.0 and RStudio version 2026.01.2.

## 3. Results

As previously described, the study population was divided into three groups, and the mean values of selected parameters were calculated for each group (Table 1). In Group I, relatively high standard deviations were observed for total cholesterol and insulin. In Group II, large standard deviations were noted for total cholesterol, LDL, HDL, triglycerides, insulin, and CRP. In Group III, elevated standard deviations were observed for LDL, HDL, triglycerides, and fasting glucose. These findings may be attributable to the presence of outliers within the respective groups. The data presented in Table 1 do not show a linear trend in all cases. Group I exhibited the most favorable metabolic profile. They had the lowest waist-to-hip ratio (WHR 0.8 ± 0.06), the lowest triglyceride levels (51 ± 1.4 mg/dL), low CRP concentrations (1.0 ± 0.1 mg/L), and relatively low insulin (8.3 ± 7.8 µIU/mL) and C-peptide levels (1.9 ± 1.8 ng/mL). Fasting glucose remained within the normal range (97 ± 2.7 mg/dL). Group II demonstrated less favorable metabolic parameters. WHR increased to 1.0 ± 0.04, total cholesterol and triglycerides were markedly higher (253 ± 7.5 mg/dL, and 158 ± 32 mg/dL, respectively), and CRP levels were substantially elevated (5.2 ± 8.4 mg/L), indicating increased inflammatory status. Insulin (12.0 ± 10.9 µIU/mL) and C-peptide (3.0 ± 2.6 ng/mL) concentrations were also higher compared to Group I. Group III showed the highest WHR (1.2 ± 0.08). Although total cholesterol was lower than in Group II, triglycerides remained elevated (114 ± 71 mg/dL). Insulin (11.0 ± 3.8 µIU/mL) and C-peptide (2.3 ± 0.8 ng/mL) levels were higher than in Group I, suggesting persistent metabolic impairment. CRP levels (2.5 ± 2.9 mg/L) were elevated compared to normal-weight individuals but lower than in the overweight group. Fasting glucose levels were comparable across groups.

The correlation analyses revealed significant associations between irisin concentration and selected metabolic parameters (Figure 1 and Table 2). The strongest correlations were observed with insulin levels (r = 0.77), the HOMA-IR index (r = 0.63), and C-peptide concentration (r = 0.71). Slightly lower, yet still positive, correlations were also observed between irisin and BMI (r = 0.54), as well as visceral fat proportion (r = 0.46). Correlations between IL-1β and the analyzed biochemical parameters were limited, with statistically significant associations observed only for BMI (r = 0.6121), WHR (r = 0.5525), and visceral adipose tissue (r = 0.48). CCL2 showed the strongest correlations with body weight-related parameters among those tested (BMI: r = 0.78; WHR: r = 0.73; visceral tissue: r = 0.61). However, unlike irisin, correlations between CCL2 and insulin, C-peptide, and HOMA-IR did not reach statistical significance. In addition, both IL-1β and CCL2 were moderately correlated with CRP (r = 0.42 and r = 0.41, respectively).

In the case of IL-6 and IL-15, correlations with the investigated variables were low and did not indicate any significant association with the examined metabolic parameters (Table 2).

The comparative analysis conducted among individuals with normal weight, overweight, and obesity revealed no differences in IL-6 (*p* = 0.79) or IL-15 (*p* = 0.51) concentrations (Figure 2). In contrast, irisin levels differed significantly between the groups (*p* < 0.0001) (Table 3). For Il-1β and CCL2, one-way ANOVA on ranks (Kruskal–Wallis test) revealed significant differences in circulating IL-1β and CCL2 levels across groups categorized by body weight status (Table 3). Overweight individuals exhibited higher irisin levels compared to those with normal weight (*p* = 0.0101), with the highest concentrations observed in the obese group relative to normal-weight participants (*p* < 0.001). The difference between the overweight and obese groups did not reach statistical significance (*p* = 0.065) (Figure 2). Additionally, results revealed significant differences in irisin between groups I–II and I–III, in IL-1β between groups I–III and II–III, and in CCL2 between all pairwise group comparisons (Figure 2).

To determine differences in irisin concentrations between groups with varying body weight, Bonferroni correction was performed. Pairwise comparisons were conducted between individuals with normal body weight (Group I; BMI: 18.5–24.9 kg/m^2^), overweight individuals (Group II; BMI: 25.0–29.9 kg/m^2^), and obese individuals (Group III; BMI ≥ 30 kg/m^2^). The results revealed statistically significant differences between Group I and Group II (mean difference = 1.6549 pg/mL; *p* = 0.0101) as well as between Group I and Group III (mean difference = 3.0886 pg/mL; *p* < 0.001). The difference between Group II and Group III did not reach statistical significance (mean difference = 1.4337 pg/mL; *p* = 0.0652). These findings indicate that irisin levels were significantly higher in overweight and obese individuals compared to those with normal body weight, with the most pronounced difference observed between Groups I and III. However, the difference between Groups II and III was not statistically significant. Overall, post hoc Mann–Whitney U tests revealed significant differences in irisin between groups I–II and I–III, in IL-1β between groups I–III and II–III, and in CCL2 between all pairwise group comparisons. No significant pairwise differences were observed for IL-6 or IL-15 (Figure 2).

All values are presented as mean differences expressed in picograms per milliliter (pg/mL), along with adjusted *p*-values for multiple comparisons.

In the final stage of analysis, which accounted for the presence of insulin resistance (Figure 3), only circulating irisin levels differed significantly between groups, with lower irisin concentrations observed in individuals exhibiting a decrease in HOMA-IR (H = 4.77, *p* = 0.029). In contrast, no significant differences were detected for IL-6 (H = 1.73, *p* = 0.19), IL-15 (H = 1.03, *p* = 0.31), IL-1β (H = 1.07, *p* = 0.30), or CCL2 (H = 0.26, *p* = 0.61) between insulin-resistance strata.

Multiple linear regression models were constructed to evaluate the independent associations of irisin, IL-6, IL-15, IL-1β, and CCL2 with metabolic outcomes (Table 4). In the HOMA-IR model, BMI was also included as a covariate to determine whether biomarker associations were independent of adiposity. In the HOMA-IR model, irisin was the only independent predictor of insulin resistance (β = 0.89, 95% CI: 0.62 to 1.16, *p* < 0.001). IL-6, IL-15, IL-1β, CCL2, and BMI were not independently associated with HOMA-IR. The model explained 71% of the variance in HOMA-IR (adjusted R^2^ = 0.68). In the BMI model, CCL2 showed the strongest independent association with BMI (β = 0.70, 95% CI: 0.33 to 1.07, *p* < 0.001), while irisin also remained independently associated with BMI (β = 0.38, 95% CI: 0.12 to 0.64, *p* = 0.004). IL-6, IL-15, and IL-1β were not independent predictors of BMI. The model explained 71% of the variance in BMI (adjusted R^2^ = 0.69).

**Figure 4:**
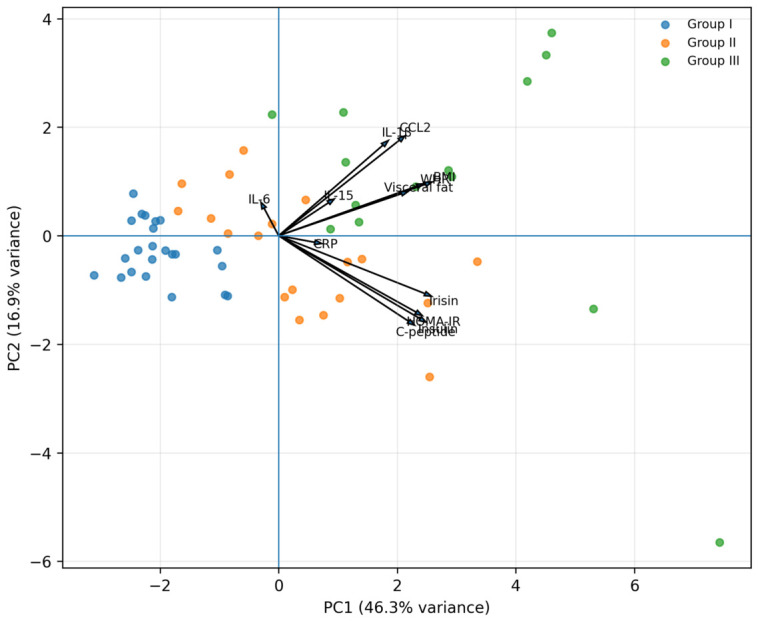
Principal component analysis of biomarkers and metabolic parameters. I—individuals with normal body weight (BMI: 18.5–24.9 kg/m^2^); II—overweight individuals (BMI: 25.0–29.9 kg/m^2^); III—obese individuals (BMI: ≥30.0 kg/m^2^).

Principal component analysis revealed two dominant components explaining the majority of variance in the dataset (Figure 4). The first principal component (PC1) accounted for 46.3% of the total variance and was characterized by strong positive loadings for HOMA-IR, insulin, C-peptide, BMI, and irisin, indicating a metabolic axis associated with insulin resistance and adiposity. The second principal component (PC2) explained 16.9% of the variance and was primarily defined by IL-1β and CCL2, reflecting an inflammatory axis. Irisin clustered closely with metabolic parameters along PC1, whereas IL-1β and CCL2 were positioned along PC2, indicating a separation between metabolic and inflammatory biomarker profiles. IL-6 and IL-15 showed relatively low loadings on both components, suggesting a limited contribution to the dominant patterns observed in this cohort.

## 4. Discussion

The aim of the present study was to assess the relationships between the concentrations of IL-6, IL-15, irisin, IL-1β CCL2, and metabolic parameters in individuals with obesity. The obtained results allow for a more detailed analysis of the roles of these associative biomarkers and their potential relevance in identifying obesity-related metabolic disturbances, although not all analyses yielded unequivocal results.

The most pronounced results were obtained for irisin, IL-1β and CCL2 whose concentrations differed significantly between patient groups according to BMI (Figure 1). Starting with irisin, both overweight and obese individuals exhibited significantly higher irisin levels compared to individuals with normal body weight, with the largest difference observed between the obese and normal-weight groups. These findings are in line with previous reports suggesting that irisin initially increases in response to worsening metabolic status in obesity [16], although the positive correlation between circulating irisin levels and obesity appears contradictory to the proposed role of irisin as a factor preventing obesity. A possible explanation is that irisin has been proposed to act as a protective factor against obesity by inducing the browning of white adipose tissue induced by physical activity, which could lead to the assumption that irisin levels rise with increasing body mass [17]. Numerous studies also highlight the role of physical activity in modulating irisin levels in individuals with varying degrees of obesity [18], although some findings indicate that despite lower physical activity in obese individuals compared to non-obese individuals, circulating irisin levels may not differ significantly between these groups [19]. This underscores the importance of accurately characterizing the study population including the distinction between fat tissue mass and lean tissue mass when interpreting the role of irisin and demonstrates that results do not always allow for straightforward interpretation. In the present study, additional analyses incorporating insulin resistance were also performed, and their results may, to some extent, support the hypothesis that irisin functions as an associative biomarker of insulin resistance and obesity (Figure 3), even prior to potential alterations reported in long-standing obesity in previous studies. Such a decrease may reflect the exhaustion of compensatory mechanisms and impaired browning of white adipose tissue [20]. The available research indicates that circulating irisin levels positively correlate with fasting glucose, insulin levels, and the HOMA-IR index [21], including in individuals with normal glucose tolerance [22], with this relationship being particularly pronounced in individuals with metabolic disorders and obesity. These studies suggest that elevated irisin levels may function as a compensatory mechanism in response to insulin resistance and excessive body mass, analogous to “leptin resistance” or “insulin resistance” phenomena [22]. It is also worth noting that some studies have reported that higher irisin levels have already been associated with increased inflammatory markers, such as tumor necrosis factor α (TNF-α), C-reactive protein, and IL-6 [23,24,25] whereas in the present study we observed significant positive correlations between irisin and BMI, insulin, C-peptide, and HOMA-IR. Interestingly, one study demonstrated an inverse correlation between irisin levels and IL-6 concentrations in obese individuals with type 2 diabetes, suggesting that higher irisin levels may be associated with a reduced inflammatory response [26]. This promising hypothesis, therefore, requires further validation in well-designed prospective and interventional studies.

In contrast to irisin, no significant differences in IL-6 concentrations were observed among individuals with normal weight, overweight, and obesity in the analyzed group. Similar findings were reported by Ashraf et al. (2018) [27], who also observed no significant differences in cytokine levels, including IL-6, between obese and non-obese patients with metabolic syndrome.

Similarly, to IL-6, no differences in the pro-inflammatory IL-15 concentrations were observed among patient groups with varying BMI [28]. However, in the analysis accounting for insulin resistance, IL-15 levels were slightly higher in the group with this disorder (*p* = 0.048). Given the modest effect size and borderline statistical significance, this finding should be interpreted cautiously. The lack of consistent associations may reflect the dual and concentration-dependent metabolic actions of IL-15 reported in experimental studies. In the retrospective study by Tarantino et al. (2021) [29], similarly to the present investigation, no association was found between IL-15 and body mass index (BMI), nor between IL-15 and waist-to-hip ratio (WHR). Furthermore, a systematic review by Silva et al. (2024) [30] pointed out the insufficient number of available studies to perform a meta-analysis, indicating a lack of consistent evidence for an association between IL-15 and markers of insulin resistance. A study published in 2020 by Nadeau et al. [15] indicated that IL-15 functions depend on its concentration. Short-term exposure to IL-15, corresponding to local muscle levels, increases basal glucose uptake and mitochondrial oxidative functions via AMPK pathway activation and the formation of respiratory chain supercomplexes. IL-15 also regulates fat metabolism and stimulates muscle development [31]. Experimental studies have shown that IL-15 supports fat mass reduction, reduces oxidative stress, and improves mitochondrial functions [11,15].

A study conducted in groups of patients with normal body weight and obesity, which analyzed the impact of increased IL-1β levels in obesity on the development of prostate cancer through the enhancement of inflammatory processes, demonstrated that obesity is associated with increased IL-1β expression in adipose tissue. This phenomenon correlates with chronic inflammation and may play an important role in the development of obesity-related comorbidities [32]. Yan et al., (2024) reported that elevated IL-1β concentrations may lead to adipocyte dysfunction through the activation of the nuclear factor κB (NF-κB) signaling pathway, which plays a significant role in the pathogenesis of intestinal inflammation [33]. Studies in murine models indicate that increased IL-1β levels activate the p38/MAPK signaling pathway in hematopoietic stem cells, thereby reducing their differentiation capacity in obesity models [33]. Furthermore, the increase in lipolytic activity induced by IL-1β occurs indirectly through enhanced leptin secretion, resulting in a significant inhibition of insulin-dependent signaling pathways [34]. Studies also suggest a role of the chemokine CCL2 in metabolic regulation. One study reported that in patients with type 2 diabetes mellitus (T2DM) with normal body weight and overweight, CCL2 concentrations were significantly higher than in control groups (*p* < 0.05) [35,36]. In the same study, individuals with obesity and morbid obesity exhibited an even more pronounced increase in CCL2 levels (*p* < 0.001), and CCL2 concentrations in obese patients with T2DM were also significantly higher compared with healthy subjects (*p* < 0.01). Similar results were reported in a meta-analysis that included 98 studies [37]. Both IL-1β and CCL2 are closely associated with the development of insulin resistance, dyslipidemia, and the progression of metabolic complications related to obesity [38,39].

In the present study, CCL2 emerged as the biomarker consistently associated with adiposity and metabolic dysfunction, showing robust group differences across groups as well as strong correlations with continuous measures of obesity and insulin resistance. CCL2 displayed the strongest associations, correlating positively with BMI, WHR, visceral adipose tissue, CRP, and indices of insulin resistance (HOMA-IR, insulin, and C-peptide). These findings align closely with the established role of CCL2 (monocyte chemoattractant protein-1) as a key mediator of obesity-induced inflammation, promoting monocyte/macrophage recruitment into adipose tissue and driving the transition toward a pro-inflammatory adipose tissue microenvironment [37]. Elevated circulating CCL2 levels have been repeatedly linked to increased visceral fat mass, insulin resistance, and cardiometabolic risk, and experimental models demonstrate that CCL2–CCR2 signaling directly contributes to macrophage accumulation and metabolic impairment in obesity [36,37].

Similarly, IL-1β showed significant positive associations with central adiposity and metabolic dysregulation, including BMI, WHR and visceral fat. IL-1β is a pivotal effector cytokine of the NLRP3 inflammasome and plays a central role in linking nutrient excess to chronic low-grade inflammation [32]. Increased IL-1β production in obesity has been shown to impair insulin signaling, reduce pancreatic β-cell function, and exacerbate systemic insulin resistance [30]. The observed gradient of IL-1β across weight groups, with highest levels in individuals with greater adiposity, supports the concept that inflammasome activation scales with fat mass and metabolic stress rather than reflecting a binary obese–non-obese state. Additionally, although IL-1β and CCL2 correlated with continuous indices of insulin resistance, their concentrations did not differ significantly between insulin-resistance strata, likely reflecting limited statistical power and the chronic nature of adipose tissue inflammation rather than short-term changes in insulin sensitivity.

Importantly, the convergence of group-based differences and continuous correlations strengthens the biological relevance of both markers. While IL-6 and IL-15 did not show consistent associations, the strong and reproducible relationships observed for CCL2 and IL-1β across independent groups highlight their potential utility as integrative markers of obesity-related inflammation and metabolic risk. Together, these results reinforce the central role of chemokine-driven immune cell recruitment (CCL2) and inflammasome-mediated cytokine activation (IL-1β) in the pathophysiology of obesity and its metabolic complications.

The multivariable analysis substantially strengthens the interpretation of the biomarker profile observed in this study. When HOMA-IR was analyzed as the dependent variable, irisin remained the only biomarker independently associated with insulin resistance after simultaneous adjustment for IL-6, IL-15, IL-1β, CCL2, and BMI. This indicates that the relationship between irisin and HOMA-IR is not merely a reflection of increased body mass or concurrent inflammatory marker concentrations. Rather, irisin appears to capture a metabolic dimension of obesity-related impairment that is not fully represented by BMI alone.

In contrast, CCL2 was the dominant independent predictor of BMI, supporting its closer relationship with adiposity-related inflammatory burden. Although IL-1β showed associations in univariate analyses, it did not remain independently associated with BMI or HOMA-IR in the multivariable models. This suggests that its apparent relationship with metabolic impairment may be partly explained by shared variance with other adiposity-linked inflammatory markers, particularly CCL2.

Taken together, these results support a biologically distinct pattern: irisin is most strongly linked to insulin resistance, whereas CCL2 is more closely associated with body mass and obesity-related inflammatory load. IL-6 and IL-15 did not show independent associations in these models, further supporting their limited value as early associative markers of metabolic impairment in this cohort.

Although the present study highlights the predominant role of irisin, potential interactions among all associative biomarkers should not be overlooked. Previous studies indicate that both IL-6 and irisin can promote adipose tissue browning and increase thermogenesis, while IL-15 supports fat reduction and enhances mitochondrial function [12,13]. It can therefore be concluded that the ultimate metabolic effect depends not on a single biomarker but on the dynamic balance between them. The absence of significant differences in IL-6 and IL-15 levels in this study, conducted on a relatively young group, may suggest that their effects manifest only over long-term metabolic processes or under specific clinical conditions, such as type 2 diabetes or non-alcoholic fatty liver disease as shown in other studies [29,40]. Furthermore, interactions among these associative biomarkers may be influenced by factors such as age, sex, fat distribution, and degree of insulin sensitivity [23,41], highlighting the need for stratified analyses in future studies. For example, while irisin may exert protective effects by inducing browning of white adipose tissue, bone metabolism [42] and improving insulin sensitivity, its interaction with IL-6 may modulate the overall inflammatory milieu, potentially enhancing or counteracting metabolic benefits depending on the systemic context [43,44]. Taken together, these considerations underscore the complexity of myokine–adipokine crosstalk in human metabolism. Understanding the temporal and context-dependent interplay among them is essential for identifying candidate associative biomarkers of metabolic dysregulation and for improving the understanding of metabolic dysregulation in obesity.

## 5. Limitations

The relatively small sample size may limit the statistical power, especially in subgroup analyses based on insulin resistance status and may also reduce the generalizability of the results. It should be noted that the relatively small sample size precludes drawing definitive conclusions regarding causal relationships or temporal associations; however, it does allow for the identification of potential associations. Although participants were required to maintain stable dietary habits and physical activity levels prior to inclusion, these factors were assessed based on self-report and were not quantified using validated instruments. The use of standardized dietary and physical activity assessment tools would improve the accuracy and reproducibility of future studies. Larger, adequately powered studies with longitudinal designs are needed to confirm the observed relationships and to determine whether the identified biomarkers can predict future metabolic deterioration rather than merely reflect concurrent metabolic status. Another significant limitation of this study is the use of BMI as the primary anthropometric index. BMI does not distinguish between fat and lean tissue mass, which is particularly important when interpreting associations with myokines such as irisin. The lack of comprehensive body composition analysis (e.g., DEXA or segmental bioimpedance) limits the ability to distinguish whether the observed associations are due to obesity, muscle mass, or their relative proportion. Additionally, inter-individual variability in biomarker levels may have influenced the observed distributions and should be considered when interpreting the results.

## 6. Conclusions

The present study demonstrates that among the analyzed associative biomarkers, irisin showed the strongest associations with increasing body mass and early metabolic alterations among the analyzed biomarkers. Significantly higher irisin concentrations were observed in overweight and obese individuals compared with normal-weight subjects, as well as in individuals with insulin resistance, suggesting that irisin may serve as an associative marker of metabolic dysregulation in obesity. The strong positive correlations between irisin and insulin, WHR, HOMA-IR, C-peptide, and BMI.

In contrast, no significant differences in serum IL-6 and IL-15 concentrations were observed across BMI categories, indicating that these cytokines may not reflect early metabolic alterations in relatively young obese individuals. However, the slightly elevated IL-15 levels in individuals with insulin resistance suggest that this cytokine may be associated with glucose metabolism regulation at more advanced stages of metabolic impairment.

Importantly, IL-1β and CCL2 exhibited distinct patterns compared with IL-6 and IL-15. Both markers showed significant increases across body weight categories and demonstrated strong positive correlations with BMI, WHR, visceral adipose tissue, and indices of insulin resistance, including HOMA-IR, insulin, and C-peptide. In particular, CCL2 displayed the strongest associations with adiposity-related and inflammatory parameters, including CRP, suggesting a potential link to obesity-driven immune activation. Despite these robust associations with adiposity and metabolic load, neither IL-1β nor CCL2 differed significantly when participants were stratified by insulin resistance status, suggesting that their circulating levels primarily reflect chronic adipose tissue inflammation rather than short-term changes in insulin sensitivity. Multivariable regression and PCA further strengthened these observations, showing that irisin is independently associated with insulin resistance, whereas CCL2 is more strongly linked to adiposity, with distinct separation between metabolic and inflammatory biomarker axes.

## Figures and Tables

**Table 1:** The characteristics of the study participants, including biochemical, hormonal, and anthropometric parameters, are summarized here. The first column indicates the weight group number, the second lists the measured parameters with their respective units, and the third shows median [Q1–Q3] calculated for each weight group.

Parameter	Group I (Normal Weight, *n* = 20)	Group II (Overweight, *n* = 20)	Group III (Obese, *n* = 11)
BMI (kg/m^2^)	22 (21–23)	28 (27–29)	34 (33–35)
WHR	0.80 (0.76–0.84)	1.00 (0.97–1.03)	1.20 (1.15–1.25)
Systolic BP (mmHg)	119 (116–122)	121 (117–125)	124 (121–127)
Diastolic BP (mmHg)	78 (74–82)	80 (77–83)	83 (80–86)
Visceral fat (level)	4 (3–5)	8 (6–10)	12 (9–15)
HOMA-IR	2.0 (1.3–2.7)	3.1 (1.8–4.4)	3.5 (2.2–4.8)
Total cholesterol (mg/dL)	193 (170–216)	253 (248–258)	204 (199–209)
LDL (mg/dL)	135 (131–139)	120 (92–148)	124 (111–137)
HDL (mg/dL)	67 (64–70)	58 (47–69)	57 (47–67)
Triglycerides (mg/dL)	51 (50–52)	158 (136–180)	114 (66–162)
Insulin (µIU/mL)	8.3 (3.0–13.6)	12.0 (4.6–19.4)	11.0 (8.4–13.6)
C-peptide (ng/mL)	1.9 (0.7–3.1)	3.0 (1.3–4.7)	2.3 (1.8–2.8)
CRP (mg/L)	1.0 (0.9–1.1)	5.2 (0–10.9)	2.5 (0.5–4.5)
Fasting glucose (mg/dL)	97 (95–99)	91 (89–93)	99 (84–114)

*n*—number of patients in the respective group; BMI—Body Mass Index; WHR—Waist-to-Hip Ratio; LDL—Low-Density Lipoprotein; HDL—High-Density Lipoprotein; TG—Triglycerides; CRP—C-reactive Protein. I—individuals with normal body weight (BMI: 18.5–24.9 kg/m^2^); II—overweight individuals (BMI: 25.0–29.9 kg/m^2^); III—obese individuals.

**Table 2:** Correlation coefficients (r) between circulating levels of interleukins (IL-6, IL-15) and irisin with selected metabolic, anthropometric, and biochemical parameters. Strong positive correlations with significance (*p* < 0.05) (r > 0.5) are marked with an asterisk “*”.

	^1^ IL-6	^2^ IL-15	Irisin	IL-1β	CCL2
^3^ BMI	0.0963	0.1918	0.5442 *	0.6121 *	0.7812 *
^4^ WHR	0.0423	0.1790	0.4197	0.5525 *	0.7304 *
Visceral tissue	0.1043	0.2040	0.4624	0.4810	0.6145 *
Systolic blood pressure	0.0593	0.3847	0.2350	0.1547	0.0875
Diastolic blood pressure	0.2140	0.1754	0.2366	0.0932	0.1455
Glucose	0.1111	0.0611	0.2041	0.1456	0.3054
Insulin	0.0871	0.1842	0.7704 *	0.3202	0.37
C-peptide	0.0878	0.1050	0.7115 *	0.3332	0.36
^5^ HOMA-IR	0.0732	0.1802	0.6332 *	0.3648	0.4
Cholesterol	0.0238	0.2482	0.3093	0.0452	0.2457
^6^ HDL	0.0573	0.0423	0.2286	0.0056	0.0454
^7^ LDL	0.0136	0.2725	0.1392	0.0458	0.1236
^8^ TG	0.0735	0.2305	0.2050	0.2365	0.0548
^9^ CRP	0.0743	0.0749	0.2112	0.4235	0.41

^1^ IL-6—Interleukin-6; ^2^ IL-15—Interleukin 15; ^3^ BMI—Body Mass Index; ^4^ WHR—Waist-to-Hip Ratio; ^5^ HOMA-IR—Homeostatic Model Assessment of Insulin Resistance; ^6^ HDL—High-Density Lipoprotein Cholesterol; ^7^ LDL—Low-Density Lipoprotein Cholesterol; ^8^ TG—Triglycerides; ^9^ CRP—C-reactive Protein.

**Table 3:** One -way ANOVA (Kruskal–Wallis test) evaluating differences in circulating levels of IL-6, IL-15, irisin, IL-1β and CCL2 between groups categorized by body weight status. The H-statistic represents the Kruskal–Wallis test value, and the *p*-value indicates the level of statistical significance. A *p*-value below 0.05 was considered statistically significant.

Biomarker	Test Results	Interpretation
IL-6	H-statistic = 0.47	No significant difference in IL-6 levels across weight groups.
	*p*-value = 0.79	
IL-15	H-statistic = 1.34	No significant difference in IL-15 levels across weight groups.
	*p*-value = 0.51	
Irisin	H-statistic = 21.14	Highly significant difference in Irisin levels between weight groups.
	*p*-value ≈ 2.6 × 10^−5^	
IL-1β	H-statistic = 26.26	Highly significant difference in IL-1β levels between weight groups.
	*p*-value ≈ 2.0 × 10^−4^	
CCL2	H-statistic = 42.06	Highly significant difference in CCL2 levels between weight groups.
	*p*-value ≈ 7.3 × 10^−6^	

**Table 4:** Multivariable linear regression analysis.

Outcome	Predictor	β	95% CI	*p*-Value	R^2^	Adj. R^2^
HOMA-IR	Irisin	0.89	0.62 to 1.16	<0.001	0.71	0.68
	IL-6	0.10	−0.02 to 0.22	0.100		
	IL-15	0.04	−0.09 to 0.17	0.524		
	IL-1β	−0.07	−0.32 to 0.18	0.586		
	CCL2	−0.04	−0.40 to 0.33	0.838		
BMI	Irisin	0.38	0.12 to 0.64	0.004	0.72	0.69
	IL-6	−0.03	−0.33 to 0.26	0.819		
	IL-15	−0.11	−0.32 to 0.10	0.315		
	IL-1β	−0.07	−0.44 to 0.31	0.723		
	CCL2	0.70	0.33 to 1.07	<0.001		

β = standardized regression coefficient. Models used complete-case analysis and HC3 robust confidence intervals.

## Data Availability

The original contributions presented in this study are included in the article. Further inquiries can be directed to the corresponding author.
